# The interactive turn in generative AI for self-harm

**DOI:** 10.3389/fpsyt.2026.1877825

**Published:** 2026-08-25

**Authors:** Li-Li Zhu, Jing Qian, Wen-Jing Yan

**Affiliations:** 1 School of Psychology, Zhejiang Normal University, Jinhua, China; 2 Department of Student Affairs, Wenzhou University, Wenzhou, China; 3 Zhejiang Provincial Clinical Research Centre for Mental Health, Affiliated Kangning Hospital, Wenzhou Medical University, Wenzhou, China

**Keywords:** classification, digital mental health, functional assessment, generative AI, multimodal interaction, non-suicidal self-injury, self-harm

## Abstract

Self-harm, particularly non-suicidal self-injury (NSSI), presents a distinctive challenge for artificial intelligence in mental health. The dominant paradigm frames AI as a classifier: ingest multimodal signals, output a risk label. This paper argues that this approach faces a principled limitation. Self-harm is functionally heterogeneous; the same behavior can serve different purposes across individuals and contexts, from affect regulation to self-punishment to interpersonal communication. A classification output can support risk stratification, but it does not by itself explain what the behavior is doing for the person, and clinical intervention often depends on that functional understanding. Generative AI opens a possible alternative path. Rather than treating AI only as a detector that reads signals and returns a verdict, AI can be conceived as an interactive system capable of sustained, function-oriented dialogue. Current evidence from conversational AI and digital mental health supports the plausibility of such interaction, but it does not establish the safety, effectiveness, or clinical utility of interactive AI for self-harm or NSSI populations. This article therefore presents the interactive turn as a conceptual and empirical research agenda, not as a validated clinical intervention. Multimodal signals may eventually enrich this dialogue, but the core shift is logical rather than technological: from one-shot classification toward iterative, supervised, safety-bounded functional assessment. The paper develops the theoretical case for this shift, distinguishes evidence-supported claims from hypotheses that require testing, and outlines a system architecture that combines a conversational agent, cognitive and safety layers, trajectory-based monitoring, escalation pathways, multimodal inputs, and human oversight.

## 1 Introduction

Self-harm is among the most pressing public mental health challenges of the present period. Non-suicidal self-injury (NSSI), defined as the deliberate destruction of body tissue without suicidal intent, affects approximately 17–18% of adolescents worldwide (1, 2). Throughout this article, NSSI refers specifically to deliberate self-injury without suicidal intent. We use self-harm as an umbrella term only when discussing literature or technologies that combine NSSI, suicidal ideation, suicide attempts, or other self-injurious outcomes. Evidence on suicidal ideation is used to illustrate adjacent AI capabilities and safety requirements; it is not treated as evidence that the same models can assess the functions of NSSI. Although NSSI is associated with increased risk of later suicide attempts, that association does not remove the clinical distinction in intent. Any interactive system focused on NSSI must assess current suicidal intent and acute risk directly. Beyond its direct physical consequences, NSSI is one of the strongest predictors of future suicide attempts, making effective detection and intervention a matter of life and death (3).

Machine learning approaches to self-harm have proliferated over the past decade. Researchers have trained models on electronic health records, social media text, neuroimaging data, and self-report questionnaires to predict who is at risk (4, 5). More recently, generative AI, including large language models (LLMs) and vision-language models (VLMs), has entered the field. The study by Jacobucci and colleagues concerns momentary suicidal ideation rather than NSSI. We cite it as an adjacent example of multimodal risk classification and the challenge of generalizing prediction across individuals, not as evidence for functional assessment of NSSI (6). These developments reflect a broader optimism that AI, particularly its generative and multimodal variants, can improve the identification of self-harm risk.

The architecture of these efforts has generally treated classification as the central task. Data flow in, a prediction flows out, and the output is a label: risk present or absent, high or low, imminent or distant. Classification estimates the probability, timing, or severity of a specified outcome and remains valuable for screening, triage, monitoring, and escalation. Yet a risk label alone is incomplete for treatment planning because it does not explain what the behavior is doing for the person or what kind of support may be needed next.

The central claim of this paper is that generative AI may support a complementary component of self-harm assessment: supervised dialogue that can help elicit the meaning and function of a person’s self-harm. This paper does not argue that this approach has already been demonstrated empirically. It argues that the interactive turn is conceptually coherent, grounded in the clinical realities of self-harm, and warrants careful development and testing alongside risk classification.

Because this is a conceptual analysis in a high-risk domain, the evidentiary status of the argument should be explicit. The functional heterogeneity of NSSI, the limitations of risk-only labels for treatment planning, and the need for human clinical judgment are supported by existing self-harm research. Evidence that conversational AI can produce therapeutic engagement or symptom improvement comes mainly from depression-, anxiety-, and psychotherapy-focused studies, not from self-harm or NSSI functional assessment. The claims that an AI agent can safely elicit self-harm functions, monitor conversational trajectories, improve linkage to human care, or reduce harm are forward-looking hypotheses that require prospective clinical testing with strong oversight.

The argument is organized around four claims. The first is that risk-only classification is insufficiently matched to the clinical reality of self-harm when used alone, because it cannot accommodate the well-established finding of functional heterogeneity. The second is that text-based generative AI offers a directionally promising complementary capability through contingent, iterative dialogue, although its feasibility for self-harm populations remains to be empirically verified. The third is that multimodal signals, including facial expression, voice, and movement, represent a natural extension of this interactive framework rather than a prerequisite for it. The fourth is that the interactive turn requires a corresponding shift in how we assess safety, from static endpoint checks to trajectory-based evaluation.

## 2 The functional heterogeneity problem

### 2.1 Self-harm is not one thing

The most consistent finding in NSSI research over the past two decades is that self-harm is not a unitary phenomenon. Nock and Prinstein’s four-function model organizes the functions of self-harm along two dimensions: automatic versus social reinforcement, and positive versus negative reinforcement. It is a widely used framework, though it has been extended and contested (7). In this model, the same act of cutting, burning, or hitting can serve any combination of four functions: automatic negative reinforcement, which involves escaping aversive internal states; automatic positive reinforcement, which involves generating feeling when numb; social positive reinforcement, which involves communicating distress or gaining attention; and social negative reinforcement, which involves escaping interpersonal demands. Large-scale studies in both community and clinical samples consistently find that automatic negative reinforcement, the use of self-harm to regulate overwhelming emotion, is the most commonly endorsed function, but substantial subgroups endorse other functions or combinations thereof (8, 9).

The functional heterogeneity of NSSI is not a niche finding. It is central to several evidence-based treatments for self-harm. Dialectical behavior therapy, mentalization-based treatment, and emotion regulation group therapy all proceed from the premise that the clinician must first understand the specific function a person’s self-harm serves before selecting an intervention strategy. Teaching emotion regulation skills to someone whose self-harm primarily serves an interpersonal communication function is unlikely to help; exploring interpersonal patterns with someone whose self-harm is driven by severe affective instability may miss the point.

Yan and colleagues have demonstrated this heterogeneity with particular clarity. Using latent profile analysis in a clinical sample of adolescents, they identified distinct functional subgroups. Some were characterized primarily by emotion regulation motives, whereas others combined self-punishment and interpersonal functions. These subgroups differed meaningfully in their clinical correlates, including depression severity, anxiety, and suicidal ideation (10). A separate line of work from the same group used network analysis to demonstrate that alexithymia, or difficulty identifying and describing one’s own emotions, connects to NSSI through specific pathways involving peer victimization and emotion dysregulation (11). These findings underscore a basic point: self-harm is embedded in a person’s emotional life, relationships, and history. It has a biography.

### 2.2 What classification contributes and where interaction adds value

Classification and interaction address different clinical questions. Classification estimates the probability, timing, or severity of a specified outcome and remains valuable for screening, triage, monitoring, and escalation. Interaction can add information about the function, context, and changeability of self-injury through iterative questioning. The proposed shift treats risk prediction as one component of a broader supervised assessment pathway. A clinically useful system may combine classification for risk stratification with dialogue for functional assessment, while allowing each component to inform safety monitoring and human review.

This is not merely a problem of predictive accuracy. A risk score can identify a clinically important state, but it does not by itself determine why the behavior is occurring or which intervention is appropriate. A person who self-harms to escape intrusive memories of trauma may need something different from a person who self-harms to feel something in the face of dissociative emptiness. A single risk score cannot resolve these distinctions. A classification model can in principle predict functional subtypes or estimate imminent versus chronic risk, but most existing AI studies in this area have optimized for risk labels rather than clinically meaningful functional outcomes.

The nomogram approach developed by Shen and colleagues provides an instructive example (12). By modeling the contributions of gender, clinical symptoms, and psychometric traits to NSSI, the nomogram produces individualized probability estimates. This is methodologically sophisticated work. Yet even a perfectly calibrated probability leaves the clinician at the bedside with the same problem: probability does not prescribe treatment. A 72% risk estimate does not tell you whether to target emotion regulation, interpersonal skills, trauma processing, or something else entirely.

**Figure 1:**
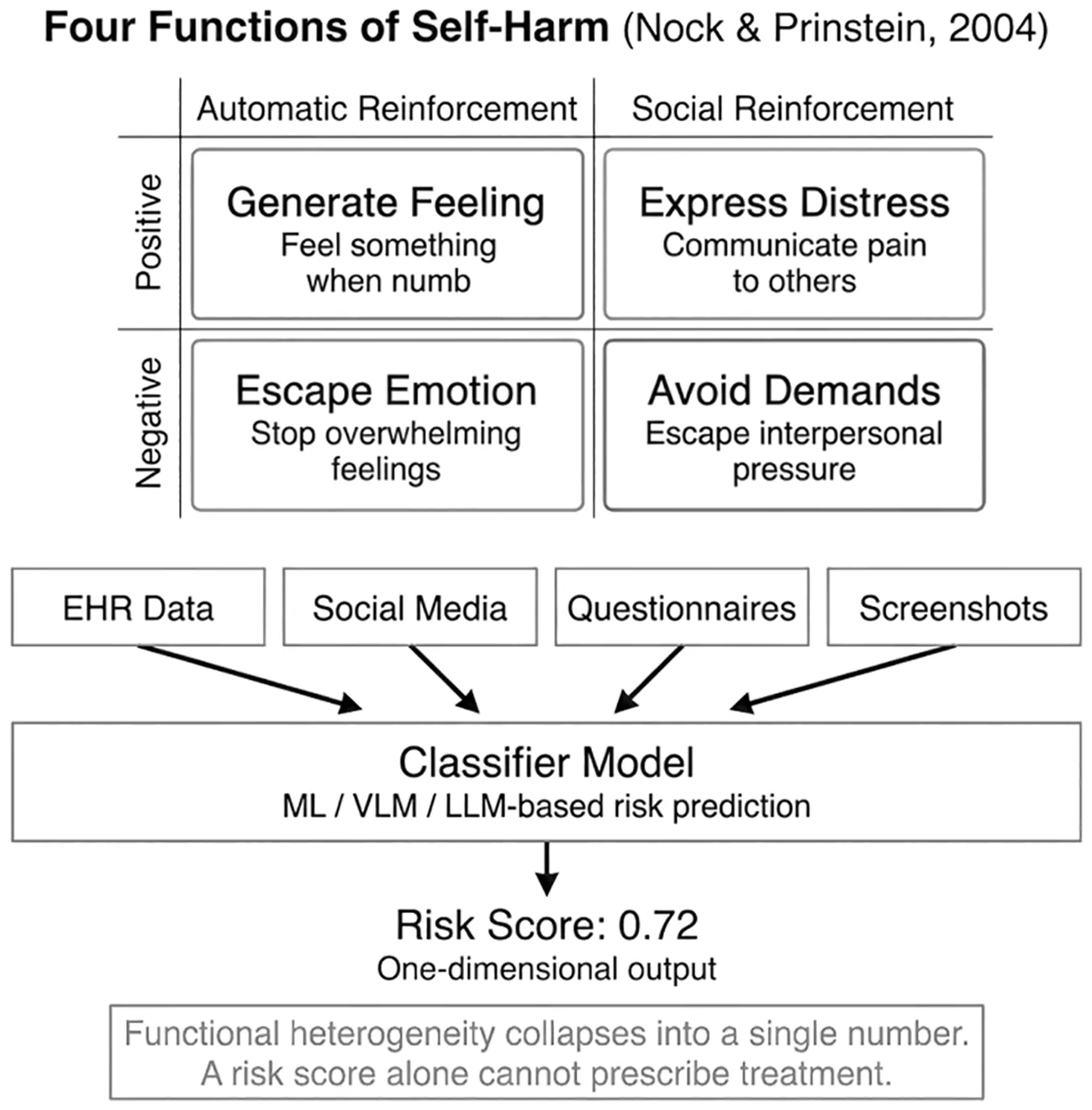
The functional heterogeneity of self-harm and its misalignment with classification-based risk prediction are illustrated in the image. The upper panel illustrates the four-function model of non-suicidal self-injury (Nock & Prinstein, 2004) (7), organized along the dimensions of automatic versus social reinforcement and positive versus negative reinforcement. The lower panel depicts a typical machine learning classification pipeline: multi-source data are fed into a classifier model that outputs a single risk score, collapsing functionally distinct behaviors into a one-dimensional output.

The study by Jacobucci and colleagues concerns momentary suicidal ideation rather than NSSI. We cite it as an adjacent example of multimodal risk classification and the challenge of generalizing prediction across individuals, not as evidence for functional assessment of NSSI (6). Screenshots, the visual content a person sees on their phone, are a novel and ecologically rich data source, and the finding that VLMs can extract predictive signal from them is important. The study also revealed that when models trained on one set of participants were applied to new individuals, performance dropped to near chance (AUC ≈ 0.50). This finding could reflect overfitting to a small sample, domain shift, or other technical factors, so it should not be taken as a general proof that classification fails. It nevertheless illustrates why prediction and interaction should be combined carefully: a model may contribute information about acute suicide risk while a supervised dialogue explores the function and context of NSSI. The contrast between functionally heterogeneous self-harm and one-dimensional classification-based risk prediction is illustrated in **Figure 1**.

## 3 Interaction as a complementary clinical logic

### 3.1 From risk stratification to function-oriented dialogue

**Figure 2:**
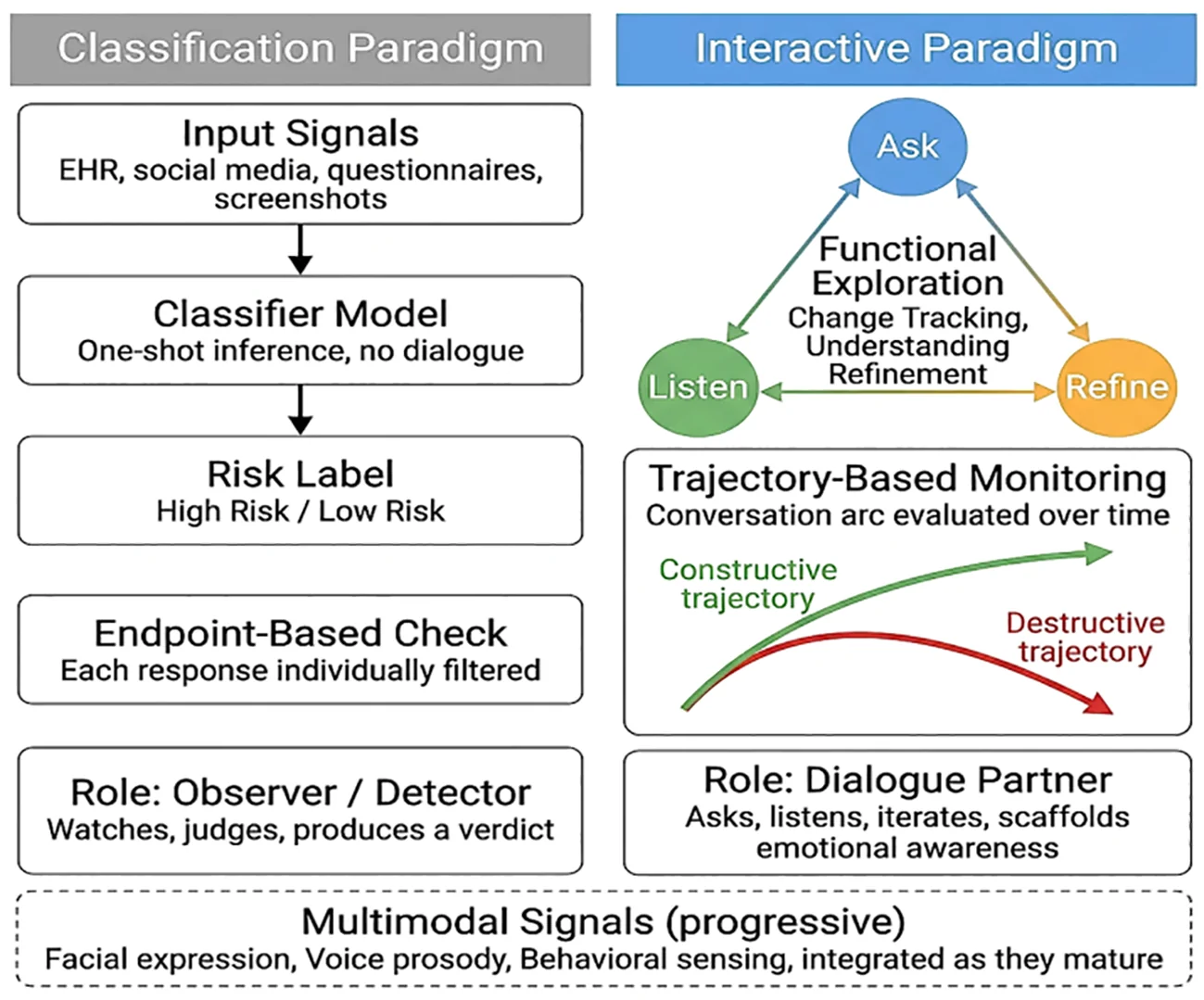
Complementary roles of classification and interaction in AI-based self-harm assessment. Classification processes multi-source signals to estimate specified risks and support screening, triage, monitoring, and escalation. Supervised interaction uses an iterative Ask–Listen–Refine cycle to explore function and context over time. Trajectory monitoring and human oversight connect the two components within a single safety-bounded architecture.

Classification and interaction are not mutually exclusive. A classifier can identify individuals whose self-harm patterns, clinical history, or digital traces suggest elevated risk; a supervised interactive system can then help explore what that risk means for a specific person. Classification provides population-level triage, while interaction may provide person-level functional understanding. If classification asks “what is the risk?”, interaction asks a different set of questions: “What is this behavior doing for you? When does it happen? What comes before, what comes after? Has anything changed?” These questions require a sequence of exchanges, not a single computation (**Figure 2**).

Generative AI, particularly LLM-based conversational agents, makes this interactive approach technically feasible as a design possibility rather than a validated clinical practice. Unlike a classifier that processes input and terminates, a generative model can sustain a conversation: it can ask a question, receive an answer, ask a follow-up based on that answer, and iterate. This capacity for contingent dialogue is the core capability that distinguishes interaction from classification. Whether this capacity can be made sufficiently safe, reliable, and clinically useful for people who self-harm remains an empirical question.

The relevance to self-harm assessment is direct but remains prospective. A conversational system could be designed to explore function through contingent questions, track change over time, and adjust its questioning when a person disengages. These capabilities are not captured by a one-shot classification architecture alone. Whether they can be implemented safely, reliably, and with clinical utility for people who self-harm remains an empirical question.

Evidence from conversational AI and digital mental health gives cautious, indirect support for the plausibility of this approach. Teferra and colleagues demonstrated that LLM responses in motivational interviewing scenarios aligned with those of human therapists on key competencies including open-ended questioning, affirmation, and reflective listening (13). Rollwage and colleagues showed that a cognitive layer architecture could enhance LLM performance in psychotherapy interactions, with improvements verified across nearly 20,000 clinical conversations (14). Randomized and meta-analytic evidence also suggests that AI-driven conversational agents can produce symptom improvement and meaningful engagement in some mental health settings: CBT-oriented chatbots have shown effects on depression and anxiety (15), a randomized trial of a generative AI chatbot reported therapeutic engagement and improvement in mental health outcomes (16), and a meta-analysis among young people found a moderate-to-large effect on depressive symptoms after adjustment for publication bias, while effects for anxiety, stress, affect, and well-being were nonsignificant (17). The generalizability of these findings to self-harm and NSSI is limited. Depression- and anxiety-focused chatbot trials typically evaluate symptom reduction, adherence, or engagement; they do not test whether an agent can conduct safe functional assessment of self-harm, avoid method-related drift, respond to imminent risk, or improve linkage to human care. The evidence therefore supports plausibility, not clinical readiness.

Conversational assessment itself is not new. Prior systems have administered structured mental-health assessments, and chatbot versions of established instruments such as the PHQ-9 have been evaluated psychometrically (18, 19). The contribution proposed here is narrower. Within AI research on self-harm, the dominant technical target has remained risk detection or stratification. We use the term interactive turn to describe a shift in emphasis from treating a risk label as the endpoint to embedding prediction within a supervised, function-oriented dialogue that can ask contingent questions, follow change over time, monitor conversational trajectory, and facilitate transfer to human care. Whether this architecture improves assessment or safety for NSSI remains to be tested.

### 3.2 What text alone can do

A natural objection at this point is that self-harm involves embodied, non-verbal dimensions that text cannot access. This objection is partly correct. We return to it in the next section. However, it misunderstands what makes the interactive turn valuable. The value of interaction over classification is not better feature extraction; it is a different epistemic stance toward the person.

Classification and interaction create different opportunities for assessment. When a person is asked about their experience and responds, they are not merely providing data; they are participating in the construction of understanding. This participatory dimension may matter for self-harm because many people experience the behavior as private, shameful, or difficult to articulate. A supervised interactive system may create conditions in which some individuals are more willing to engage, but this remains a hypothesis. Participation also creates design obligations: consent, user control, transparency about the system’s limits, and clear arrangements for involving clinicians or caregivers when this is clinically appropriate and ethically justified.

Empirical support for this claim comes from the alexithymia literature. Alexithymia is one of the most consistently replicated correlates of NSSI. A meta-analysis by Greene and colleagues found moderate associations between alexithymia and NSSI, with the difficulty-identifying-feelings dimension showing the strongest relationship (20). Cerutti and colleagues demonstrated that this difficulty operates partly through impaired emotion regulation (21). More recently, Yenen Menderes and colleagues showed that emotion dysregulation, impulsivity, and low self-esteem partially mediate the alexithymia–NSSI pathway (22). The clinical implication is clear: many people who self-harm struggle to name what they feel. An AI that asks “Can you describe what you were feeling right before it happened?” and then helps the person find words by offering possibilities, checking understanding, and refining its response performs a qualitatively different function from an AI that extracts features from text and outputs a score. The former scaffolds emotional awareness; the latter bypasses it.

Text-based interaction has limits. People in acute crisis may be unable or unwilling to type. Some individuals will find text communication alienating, evasive, or insufficient for conveying embodied distress. Text also provides no direct access to facial expression, voice prosody, injury severity, or environmental risk. The argument is therefore not that text is enough for clinical self-harm care. It is that text-based dialogue offers a researchable starting point for function-oriented interaction, provided that safety boundaries, escalation protocols, and human oversight are built into the system from the outset.

Designing such dialogue requires clinical judgment, not just engineering skill. The agent must know when to pursue a functional line of questioning and when to step back. It must recognize affect in text, including short responses, deflections, and topic shifts, and respond appropriately. It must handle disclosure of imminent risk with clear protocols for escalation. The cognitive layer architecture proposed by Rollwage and colleagues offers one template: a structured reasoning module that sits between the language model and the user, constraining outputs to clinically appropriate channels while preserving the flexibility that makes interaction valuable (14). For self-harm specifically, such a layer might enforce a sequence that begins with functional exploration, moves toward identification of alternatives, and continuously monitors for escalation signals without rigid scripting that makes the interaction feel mechanical.

## 4 Multimodal horizons

### 4.1 Why non-verbal signals matter for self-harm

The interactive turn does not require multimodal sensing, but it is enriched by it. For self-harm specifically, there are reasons to believe that non-verbal signals carry clinically relevant information that text alone cannot capture.

The alexithymia evidence reviewed above provides one rationale. If a person struggles to put their emotional state into words, then non-verbal channels, including facial expression, voice prosody, and body movement, may carry information about affective states that the person cannot verbalize. A face that flattens when a certain topic arises, a voice that drops in volume, and a hand that begins to tremor are signals that a human clinician registers and integrates into an ongoing assessment. An AI system that can perceive and integrate these signals alongside language is closer to the clinical ideal than one restricted to text.

More broadly, the NSSI literature indicates that emotion-related difficulties cannot be reduced to isolated signal detection. Emotion dysregulation is robustly associated with NSSI (23), and multimodal emotion-recognition research more generally shows that affective signals are context-sensitive and shaped by modality, dataset, and real-world conditions (24, 25). This supports a narrower point: if emotional signals are used in AI systems for self-harm, they should be interpreted as prompts for contextual inquiry rather than as stand-alone clinical evidence.

Beyond emotion, the broader digital phenotype literature suggests that behavioral patterns detectable through smartphone sensors, including movement, social interaction frequency, and sleep disruption, carry information about mental state trajectories that may precede self-harm episodes (26). These signals are not diagnostic in themselves, but they can serve as contextual information that enriches an ongoing interactive assessment. A conversational agent that knows a person’s sleep has been disrupted for three nights might ask different questions than one operating without that context.

### 4.2 The current state and the path forward

Current multimodal AI has clear limits. In controlled laboratory settings, multimodal emotion recognition systems achieve respectable accuracy. In real-world conditions, performance drops considerably. Zhang and Lu reported that a state-of-the-art multimodal emotion recognition system achieved only 61% accuracy for valence and 60% for arousal on a naturalistic dataset, far below what would be required for clinical deployment (27). The challenges are well documented: emotion categories are subjective and culturally variable, training datasets are biased toward certain demographics, and performance degrades sharply when moving from posed expressions in laboratory lighting to spontaneous expressions in uncontrolled environments (24, 25).

These limitations are real, and they are the reason this paper does not treat multimodal sensing as a prerequisite for the interactive turn. Text-based conversational systems are already available and can be evaluated as research platforms for function-oriented dialogue; this does not mean they are ready for autonomous clinical use with people who self-harm. Their safety, effectiveness, acceptability, and escalation procedures must be established before deployment in self-harm populations. As facial expression recognition, voice analysis, and behavioral sensing improve, these modalities can be integrated as additional channels of information flowing into an already-defined interactive architecture. The architecture does not need to assume that every signal is reliable; it should specify how uncertain signals are weighted, questioned, and overridden by human judgment.

This staged approach has a practical advantage. A system that depends on fully mature multimodal AI cannot be evaluated until those technologies are clinically reliable. A text-first system can be prototyped and tested under controlled, clinically supervised conditions while the multimodal components remain optional and investigational. The interactive turn is therefore a present research possibility, not a present claim of clinical effectiveness. Its value will be determined by whether such systems can be shown to improve functional understanding and help-seeking without increasing risk.

A further distinction is helpful. Multimodal signals can be gathered actively, when the person is asked to speak, show their face, or perform a task, or passively through ambient sensing that does not require explicit engagement. The interactive turn favors the active route. When a person chooses to speak into a microphone or show their face on camera as part of a conversation, the act of doing so is itself a communicative gesture. It is part of the interaction, not data extracted from it. Passive sensing, by contrast, risks reproducing the surveillance dynamic that the interactive turn seeks to replace. The design principle is simple: multimodal inputs should flow through the interaction, not around it.

## 5 Safety and ethics in the interactive paradigm

### 5.1 The risks of interaction

Interactive AI is not inherently safer than classification AI; in some respects, it introduces new risks. Dohnány and colleagues have described the phenomenon of “technological folie à deux,” in which AI chatbots and users mutually reinforce maladaptive patterns (28). A person who uses self-harm to regulate emotion might, in conversation with an AI, receive responses that inadvertently validate or normalize the behavior. A person with rigid beliefs about their unworthiness might find those beliefs reflected back. The interactive nature of the exchange can become the mechanism of harm if the system lacks the clinical judgment to recognize and interrupt pathological patterns.

For self-harm specifically, there are domain-specific risks. Online communities dedicated to self-harm are known to facilitate social contagion, normalization, and sharing of methods (29). An interactive AI that is insufficiently guarded could, in principle, function similarly if it fails to recognize when its responses are interpreted as permission or encouragement. The phenomenon of “pro-ana” and “pro-self-harm” content illustrates that people with these behaviors can form communities that reinforce rather than reduce the behavior. An AI that learns from user interactions without explicit safety constraints risks drifting in the same direction.

To make the risk assessment more concrete, we can identify at least four distinct failure modes for an interactive AI engaged in functional assessment of self-harm. First, validation seeking: a person may approach the interaction not to explore alternatives but to receive implicit permission to continue self-harming, and the AI’s neutral, accepting stance may be experienced as endorsement. Second, functional fixation: by helping a person articulate what self-harm does for them, the system may inadvertently strengthen the person’s conviction that self-harm is the only viable strategy, particularly if the AI fails to introduce alternatives at the right moment. Third, method specification: a dialogue that begins with functional exploration could drift toward detailed discussion of methods, including how, where, and with what, and the AI may not recognize this drift as harmful if its safety filters are triggered only by explicit method-related keywords. Fourth, delay of human care: a person who finds the AI interaction sufficiently satisfying may postpone or forgo seeking human clinical contact, particularly in settings where accessing human care requires effort, courage, or resources.

These failure modes are not hypothetical. Each has analogues in existing clinical and online contexts. The challenge is that an interactive AI may produce them at scale, with consistency, and without the corrective influence of a human clinician who can read the situation and intervene. Mitigating them requires design choices that go beyond content filters: explicit guardrails that interrupt validation-seeking patterns, protocols for shifting from functional exploration to alternative generation, hard boundaries around method discussion, and active encouragement of human help-seeking at regular intervals. None of these mitigations is trivial to implement, and all require empirical validation before deployment with vulnerable populations.

### 5.2 Trajectory-based safety assessment

The standard approach to AI safety in mental health is endpoint-based: does the system’s output contain harmful content? This approach, while necessary, is insufficient for interactive systems. A single exchange may appear benign while a sequence of exchanges leads a person toward a worse state. Morrin and colleagues have argued for a shift from endpoint-based to trajectory-based safety assessment: evaluating not whether each individual response passes a safety check but whether the arc of the conversation moves the person toward or away from well-being (30).

This framework maps naturally onto the interactive approach to self-harm. A conversation that begins with a person describing self-harm in functional terms, “It’s the only way I can stop the thoughts,” and gradually moves toward naming alternative strategies, however tentatively, is on a constructive trajectory even if individual exchanges contain descriptions of self-harm that would trigger endpoint-based filters. Conversely, a conversation that begins with apparently benign small talk and drifts toward increasingly detailed descriptions of method, without any countervailing exploration of alternatives, is on a destructive trajectory even if no single exchange crosses a safety threshold.

Trajectory-based assessment requires that the AI system itself be capable of tracking conversational arcs and recognizing when a dialogue is spiraling toward or away from risk. This is a challenging technical requirement, and it remains an unsolved research problem. Methods for real-time trajectory detection in clinical conversations do not yet exist at the reliability needed for high-stakes applications. What the interactive paradigm offers is not a solution but a framework: an AI that sustains a conversation can, in principle, also monitor its own conversational trajectory, whereas a classifier has no trajectory to monitor. Realizing this capability will require computational methods that combine natural language processing, longitudinal modeling, and clinician-informed annotation. These methods are not yet available.

### 5.3 The safety advantage of engagement

There is a counterintuitive safety argument for interaction. People who self-harm often avoid clinical contact. The behavior is stigmatized, disclosure is frightening, and many individuals fear involuntary hospitalization or loss of autonomy. A classification system that silently monitors and flags risk may be perceived as surveillance, reinforcing the very avoidance it seeks to overcome. An interactive system that positions itself as a conversational partner rather than a detector may, for some individuals, lower the barrier to engagement. If a person is willing to talk to an AI about their self-harm when they would not talk to a human clinician, that conversation, even an imperfect one, is a point of contact that otherwise would not exist.

This argument must be made carefully. It is not a justification for replacing human care with AI. The interactive turn is most defensible when positioned as a bridge to human care with explicit mechanisms for facilitating that transition, and most vulnerable when deployed as a standalone alternative. The distinction between engagement as a bridge and engagement as a replacement is not rhetorical; it is the line between a system that extends the clinical network and one that erodes it.

### 5.4 Participation, co-production, and human oversight

Recent work on suicide-prevention technology broadens this point by calling for a shift from prediction and surveillance toward participation, co-production, and relational care. Nguyen argues that digital suicide-prevention tools should involve caregivers in design, use, and governance, so that technology becomes a shared instrument of care rather than a system that merely watches for risk (31). This perspective is aligned with the interactive turn, but it also extends it. Functional assessment cannot be reduced to a private exchange between a user and an AI system. For many adolescents and young adults, safety depends on a wider care ecology: trusted caregivers, clinicians, schools, crisis services, and governance processes that determine how information is used and when escalation is justified.

**Figure 3:**
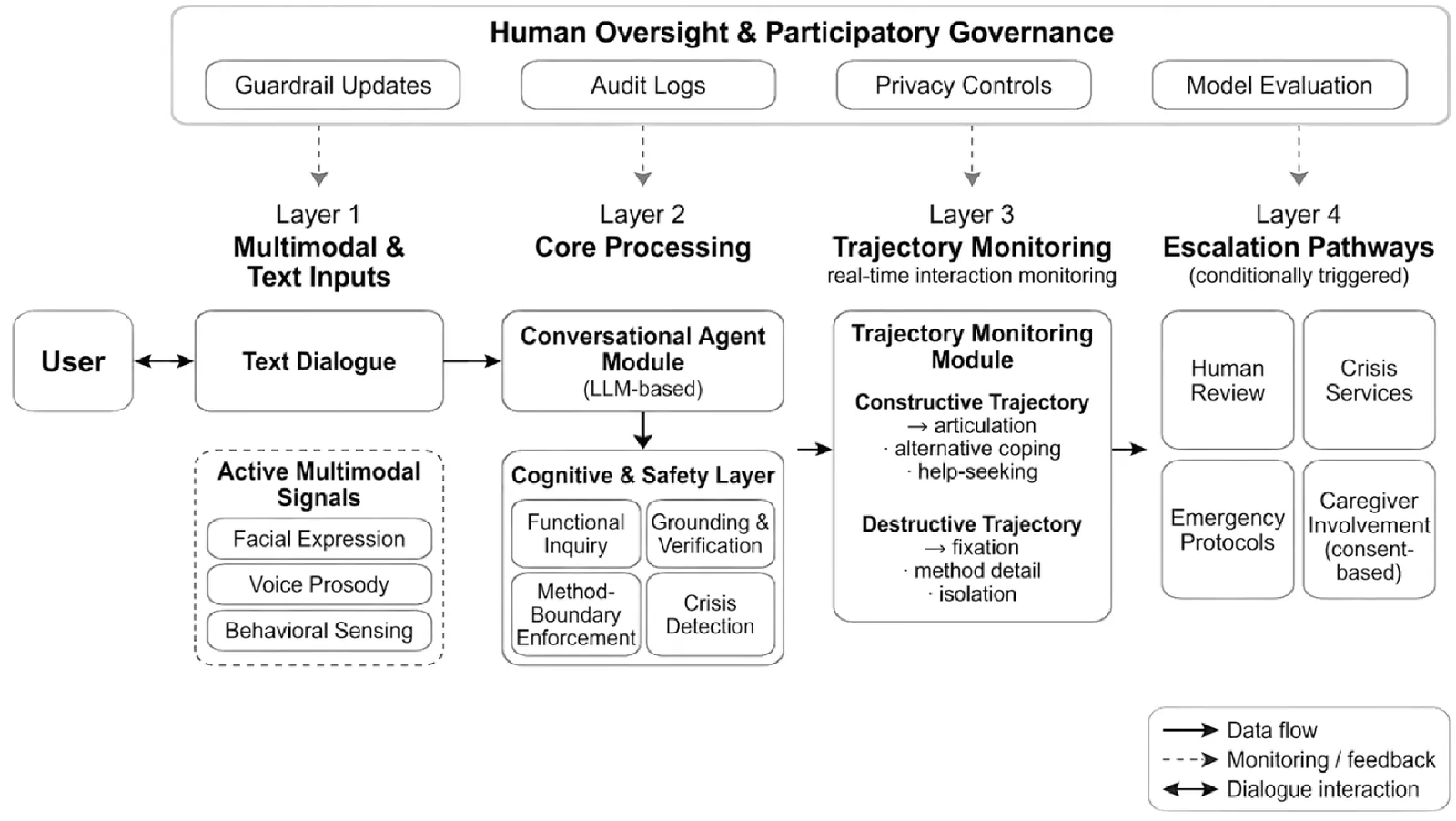
Conceptual architecture for an interactive generative-AI system for self-harm functional assessment.

A clinically credible implementation would therefore require an architecture that makes human participation and accountability visible (**Figure 3**). The conversational agent should be only one component. User-facing responses need to pass through a cognitive and safety layer that constrains functional inquiry, prohibits method elaboration, and detects imminent risk. A trajectory-monitoring module should assess whether the conversation is moving toward articulation, alternative coping, and help-seeking, or toward fixation, method detail, and isolation. Escalation pathways should connect risk states to human review, crisis services, emergency protocols, and, when consented and legally permissible, caregiver involvement. Human oversight and shared governance should feed back into model evaluation, audit logs, privacy rules, and updates to safety thresholds. In this architecture, the interactive system is not a replacement for relational care; it is a monitored interface within a larger care network.

### 5.5 Transparency and the question of regulation

An interactive AI that explores the function of a person’s self-harm is doing something clinical. It is not merely providing information or tracking mood. It is engaging with a behavior that carries risk of serious harm. This raises questions about what such a system should disclose to users and how it should be regulated.

On disclosure, the interactive turn suggests a principle of radical transparency: the system should make clear that it is an AI, that it does not replace clinical care, and that its understanding of the person’s self-harm is partial and provisional. This is not merely an ethical nicety. A person who believes they are talking to a human or to an AI that truly understands them may disclose more than they intended, with consequences they did not anticipate. Transparency about the system’s nature and limits is a precondition for informed engagement.

Regulatory status should therefore be described cautiously. In the United States, the FDA’s Clinical Decision Support Software guidance distinguishes non-device clinical decision support from device software functions by criteria including the intended user, the type of information analyzed, the nature of the recommendation, and whether a health care professional can independently review the basis for the output (32). A supervised system that elicits self-harm functions through dialogue might, in some implementations, resemble decision support or communication support, whereas functions that analyze patient-specific signals to recommend diagnosis, treatment, or urgent action could fall closer to device software oversight. In the European Union, the Medical Device Regulation governs the placing on the market and putting into service of medical devices, while the Artificial Intelligence Act adds a risk-based framework for AI systems, including high-risk systems in specified health and safety contexts (33, 34). We therefore do not treat the interactive turn as a settled regulatory category. Its classification would depend on intended use, target users, outputs, data sources, degree of automation, and the extent to which clinicians can review, override, and document system recommendations. Future systems should be designed with regulatory review, clinical governance, and post-deployment monitoring from the outset.

## 6 Discussion

We have argued that classification and interaction can make complementary contributions to AI-based self-harm assessment. Classification can support risk stratification, screening, triage, monitoring, and escalation; supervised interaction may help explore the function and context of self-harm over time. The interactive turn therefore does not replace risk prediction. It proposes embedding prediction within a broader safety-bounded pathway that includes function-oriented dialogue, trajectory monitoring, escalation pathways, and human oversight. NSSI and suicidal thoughts and behaviors are clinically distinct because intent matters, although they are longitudinally related and may share some functions and risk factors (35). An interactive system designed around NSSI must therefore assess suicidal intent and acute risk directly while avoiding the assumption that NSSI is itself a suicide attempt.

This reframing carries implications for research, development, and clinical practice. For research, evaluation should move beyond classification accuracy without jumping directly to claims of clinical efficacy. Early studies should test whether interactive systems can elicit functional information that clinicians judge useful, whether users experience the dialogue as acceptable and trustworthy, whether escalation occurs appropriately, and whether the interaction increases rather than delays help-seeking. Metrics grounded in interaction, such as functional articulation, movement toward alternative coping, successful handoff to human support, and absence of harmful conversational drift, should be evaluated alongside predictive performance for this specific task. Development priorities therefore include designing supervised conversational architectures that support functional exploration, trajectory monitoring, and human review.

This paper has limits. First, it is a conceptual analysis; the interactive turn requires empirical testing before its clinical validity can be established. The central claims that interactive AI can elicit functional information about self-harm, that trajectory-based safety monitoring is feasible, that the approach is acceptable to users, and that it can improve linkage to human care are hypotheses, not demonstrated facts. Second, the conversational AI evidence reviewed here is indirect. Randomized trials and meta-analyses in depression, anxiety, and psychotherapy-related settings support the plausibility of therapeutic engagement, but they do not establish safety, effectiveness, or clinical utility for functional assessment of self-harm or NSSI. Third, current LLMs carry well-documented limitations in consistency, reasoning depth, factual reliability, and boundary maintenance that constrain their readiness for high-stakes clinical applications. The interactive turn does not dismiss these concerns; it requires that they be addressed through cognitive and safety layers, clinical supervision, conservative escalation rules, and prospective validation. Fourth, the evidence base draws primarily on Chinese adolescent samples for NSSI heterogeneity data and on Western, English-language populations for LLM capability evidence. Cross-cultural variation in self-harm functions, emotional expression norms, caregiver roles, and help-seeking behaviors means that an interactive AI trained predominantly on Western conversational data may not generalize. Fifth, interactive functional assessment generates narrative data far more revealing than risk scores, raising privacy, consent, caregiver access, and data governance questions that require dedicated empirical and ethical work.

Self-harm is a complex, personal, and often deeply private behavior. The idea that a machine could understand it is properly met with skepticism. The interactive turn does not claim that machines understand self-harm as humans do, and it does not claim that current systems are ready to conduct autonomous clinical assessment. It claims something narrower: that generative AI may be designed to support the process of understanding by asking rather than assuming, listening rather than labeling, and staying within a supervised conversation rather than delivering a verdict and departing. Whether that design can be made safe and clinically useful is the work that remains.

## Data Availability

The original contributions presented in the study are included in the article/supplementary material. Further inquiries can be directed to the corresponding author.
